# Protective Effects of a Nano-Formulation of Curcumin against Cuprizone-Induced Demyelination in the Mouse Corpus Callosum

**DOI:** 10.22037/ijpr.2020.112952.14033

**Published:** 2020

**Authors:** Mahsa Motavaf, Majid Sadeghizadeh, Sadegh Babashah, Leila Zare, Mohammad Javan

**Affiliations:** a *Department of Molecular Genetics, Faculty of Biological Sciences, Tarbiat Modares University, Tehran, Iran.*; b *Department of Physiology, Faculty of Medical Sciences, Tarbiat Modares University, Tehran, Iran.*; c *Department of Stem Cell, Royan Institute, Tehran, Iran.*

**Keywords:** Multiple sclerosis, Myelin, Curcumin, Nano-carrier, Oligodendrocyte

## Abstract

Multiple sclerosis (MS) is a demyelinating disease of the central nervous system (CNS), characterized by neuroinflammation, oligodendrocytes (OLs) loss, and demyelination Curcumin, a natural phenolic substance, has been shown to have significant therapeutic properties in various neurodegenerative diseases, including MS. In our laboratory by loading curcumin in dendrosome nanoparticles we improved its solubility and bioavailability. Our previous study showed anti-inflammatory and anti-oxidative effects of dendrosomal nano-curcumin (DNC) in experimental autoimmune encephalomyelitis (EAE) model of MS. Here, by using a toxic demyelination model, induced by cuprizone (CPZ), we investigated the protective effect of DNC on oligodendroglial lineage cells (OLLC) and myelin preservation in context of acute demyelination. CPZ is a copper chelator, thus its intake reduces the mitochondrial activity, activates oxidative stress response, leading to specific OLs death, due to their high-energy consumption. We also evaluated DNC effect on activation of astrocytes and microglia, which are enriched in both MS and CPZ demyelinated lesions. Our results demonstrated that DNC treatment protected Oligodendrocyte lineage cells (OLLCs) against CPZ toxin. Besides DNC treatment suppressed accumulation of astrocytes and microglia in CC of CPZ-fed mice, compared to PBS treated onse. Moreover, DNC treatment lead to higher index of luxol fast bluefast blue (LFB) and myelin-specific proteins, myelin basic protein (MBP) intensity in the corpus callosum (CC), as indicators of myelin content. These results suggest a potent pleiotropic therapeutic efficiency for DNC for protection of myelinating cells, possibly via suppression of astrocytes and microglia.

## Introduction

Central nervous system (CNS) myelination is an essential developmental phenomenon of the vertebrates. The myelin sheaths provide electrical insulation to axons, thereby facilitating the transmission of electrical impulses called action potentials. Oligodendrocytes (OLs) are the sole cells responsible for CNS myelination. Multiple sclerosis (MS) is an autoimmune disease of CNS, characterized by inflammation, demyelination and axonal damage. Although the precise pathogenesis of MS has not been fully revealed, OLs death followed by demyelination plays crucial roles in its onset. In their eminent work, Lucchinetti *et al.* (1) subtyped MS lesion into 4 different patterns. In patterns 1 and 2, lesions seem to be mediated by autoimmunity, while both pattern 3 and 4 lesions are presumed to be a primary oligodendrogliapathy. Even though the precise molecular function of each of these patterns can be different, they share a common feature; that is, tissue injury as consequence of reactive oxygen production and mitochondrial dysfunction. Since OLs are high energy consumer, they are very sensitive to oxidative stress. Cuprizone (CPZ), a chemical copper chelator and mitochondrial toxin, produces mitochondrial stress and triggers the local immune response in corpus callosum (CC), when administered through food. Biochemical and cellular responses against CPZ, ultimately result in selective loss of OLs, microglia accumulation and gliosis in CC. It is likely that reactive oxygen species (ROS) production by damaged mitochondria is one of the major causes for OLs dysfunction and persistent demyelination. Remarkably, several aspects of the histological pattern induced by CPZ are similar to demyelinated MS lesions (2-4). Thus, this model is an appropriate pharmacological model to explore myelin protection and regeneration after drug intervention.

Curcumin, a natural, lipid-soluble compound extracted from plant *Curcuma longa* is a highly pleiotropic molecule with many pharmacological activities (5). Studies of cellular and animal models have showed beneficial effects of curcumin in neurodegenerative disorders including Alzheimer disease, Parkinson disease, MS, and Huntington’s disease (6-9). Despite immense therapeutic effectiveness of curcumin, its poor bioavailability and rapid clearance have hampered its widespread therapeutic application. To overcome this issue, several approaches using adjuvants, liposomes, and nanoparticles have been adapted (10, 11).

In our laboratory we used dendrosome, a neutral, amphipathic and biodegradable nano-material as carrier for delivery of curcumin (12-14). Our findings have indicated that formulation of curcumin as DNC improves its solubility and bioavailability, beside, facilitates its cellular uptake (15-21).

On the basis of immunomodulatory and anti-oxidative properties of curcumin, in our previous study we evaluated the therapeutic effects of DNC in experimental autoimmune encephalomyelitis (EAE), an immune-mediated model of MS. We indicated that DNC postponed EAE development and attenuated its clinical scores, mainly through immunomodulatory and anti-oxidative stress mechanisms. Accordingly, here by using CPZ model, we investigated the effect of DNC on oligodendroglial cell survival, astrocyte reactivity, microglial population and myelin preservation, during the course of acute CPZ treatment. DNC was prepared according to previous method, developed by our laboratory. Demyelination was induced by feeding the animals with 0.2% CPZ for 6 weeks. Experimental groups were included, intact animals and those that received DNC or PBS during CPZ fed period. The intensity of myelin, as well as number of oligodendroglial cells, astrocytes and microglia was compared among different groups. Besides, as reports about integrity of blood-brain barrier (BBB) fallowing CPZ intake is controversial (22), we also investigated the ability of DNC to cross the BBB of intact mice *in-vivo* and *ex-vivo*.

## Experimental

*DNC preparation*

DNC was prepared according to optimized protocol in our laboratory (19). Briefly, dendrosome (Polymeric micelle OA400 carrier) was synthesized by esterification of oleoyl chloride (0.01 mol) and polyethylene glycol 400 (0.01 mol) at 25 °C for 4 hours, in the presence of triethyl amine (0.012 mol) and chloroform as solvent. Then, by filtering the product, organic phase and trimethylamine hydrochloride salt were separated. Chloroform was eliminated by evaporation from dendrosome in vacuum oven at 40 °C for 4 h. In order to synthesize DNC, curcumin with 95% purity (purchased from Merck KGaA (Darmstadt, Germany) were co-dissolved in 5 mL acetone and then, this solution was added into 5 mL PBS while being stirred constantly. According to our previous findings, we chose ratios of dendrosome/curcumin are 25:1 as best weight/weight ratio (20). Gou et al’s protocol (23) was used for loading dendrosome nanocarriers with curcumin molecules. Finally, the acetone was eliminated and sterilization of curcumin/dendrosome micelle solution was done using a 0.22 μm syringe filter (23). The prepared DNC was stored in dark condition at 4 °C (20). 

*In-vivo imaging*

As nano-formulation of curcumin has auto-fluorescent property (24), with an emission peak at ~550 nm under UV light (25), we investigated its ability to cross intact BBB using and *in-vivo* imaging system (UVITEC Ltd, Alliance Q9, Cambridge, UK). Intact mouse was received single dose of DNC (12.5 mg/kg), intraperitoneally. After half, one and two hours, the mouse was anesthetized and the hair on top of its skull was removed before being placed in apparatus darkroom. UV-excitation beam was set and emission wavelength was monitored at 530-580 nm. After the last *in-vivo* imaging*, *mouse was sacrificed; the brain was removed from the skull immediately and placed into the imaging system darkroom at the identical position as scanned live animal. The brain was excited by UV emission and then analyzed for fluorescence detection (26). Along with DNC treated mice, same *in-vivo* imaging approach was performed in control mice.

*Animals and treatments*

Seven-week old male C57BL/6 mice were purchased from Pasteur institute (Tehran, IRAN). After 1 week of acclimation, to experimentally induce acute demyelination, the mice were fed 0.2% CPZ-containing (Sigma Aldrich) chow, for 6 weeks. During period of CPZ feeding, the mice we divided randomly to receive i.p. injections of DNC (12.5 mg/kg/day) (9) or PBS. At the end of CPZ feeding, the animals were anesthetized by chloride dehydrate and trans-cardially perfused with PBS followed by 4% paraformaldehyde. Then, the brains were harvested, sectioned, and analyzed. All procedures were performed in accordance with the NIH guidelines for research involving laboratory animals. The procedures were approved by Tarbiat Modares University, Tehran, Iran Committee of Ethics in Research.

*Assessment of myelin density by luxol fast blue (LFB)*

The removed brains were post-fixed in 4% paraformaldehyde (overnight in 4 °C), and sectioned as 0.6mm coronal sections using a cryostat device. As CPZ treatment leads to greatest extent of demyelination in the caudal regions of CC (27, 28), 9 caudal (C; Bregma −1.5 to−2.0 mm), coronal slices from CC of three mice per treatment group were examined. The tissue sections were double-stained with hematoxylin and eosin (H&E) and LFB following the previously describe protocol (29). Briefly, the brain tissues were immersed in 0.1% LFB solution (British Drug House, UK) for 2 h, at 60 °C. Then, excess dye was removed with 95% ethanol. Sufficient contrast was obtained by rapid immersion of slides in 0.05% lithium carbonate and 70% ethanol until the gray matter became colorless. In order to counterstain the sections, after washing them with double-distilled water, they were steeped in 0.1% cresyl violet for 4 minutes at 37 °C. Subsequently each slice was differentiated in 95% ethanol for 5 min. Following that H&E staining was applied by staining with Harris Hematoxylin for 4 min and then clearance of sections in xylene. After washing and counterstaining, the sections were cover-slipped Entellan (Merck Chemicals, Germany). They were then viewed under an optical microscope. The degree of myelination was quantitatively evaluated by LFB intensity correlation analysis using ImageJ software.

*Immunohistofluorescence staining (IHF)*

IHF staining was performed as previously described (30). Briefly sections were incubated in triton-X100 (0.2%), blocked using 10% NGS for 1h, and then were incubated overnight in primary antibody at 4 °C. The day after, sections were rinsed with ice-cold PBS-tween and then incubated with secondary fluorescence-labeled antibody for 1 h at room temperature (RT). The sections were then washed in ice-cold PBS-tween, followed by 1 min DAPI staining. The sections from all mice at each time point were immunostainings at the same time. Semi-quantification of the intensities was evaluated using ImageJ software while ensuring consistency of processing, exposure to light and image acquisition. The fluorescence signal profile was normalized by the background intensity. The degree of myelination was evaluated by IHF for MBP, marker of myelin production. The intensities were evaluated using ImageJ software (version 1.50e; U.S. National Institute of Health). The fluorescence signal profile was normalized by the background intensity.

Quantification of OLLC, astrocytes, and microglia was performed by manually counting the number of positive olig2, GFAP, and Iba1 cells in the CC, respectively (Table 1)

*Statistical analysis*

GraphPad Prism software (version 6.07, Inc. San Diego, CA, USA) was used to perform statistical analyses. Continuous numerical data were expressed as mean ± standard error of the mean. Differences between three or more groups were analyzed by ordinary one-way ANOVA followed by Tukey’s multiple comparison tests. Differences were considered statistically significant when at least a 95% confidence level was achieved (*p *< 0.05). For all graphs statistical significance is indicated by *p < 0.05; **p < 0.01; ***p < 0.001 and ****p < 0.0001.

## Result and Discussion

*DNC ability to cross the intact BBB*

**Figure 1:**
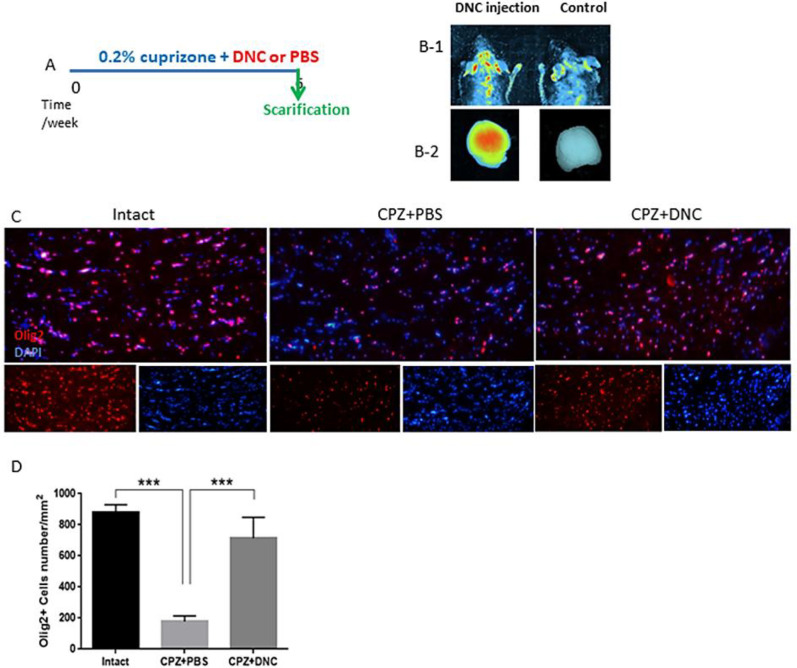
DNC treatment exhibited protective effect on OLLCs. A. A schematic image of experimental design. B) *In-vivo* imaging results showing that DNC is able to pass through intact BBB of normal mice (right). Under the same imaging conditions the level of fluorescence intensity in control mice was not indicated C) Representative image of brain section IHF by OliG2, maker of OLLCs. D) Analyzing the number of Olig2^+^ cells in intact, CPZ + PBS and CPZ + DNC showed that the number of OLLCs was significantly lower in PBC treated group compared to intact and DNC treated mice. However, no significant differebce was observed in the number of OLLCs in DNC treated group. n = 27 brain sections from 3 mice, per experiment group. Values are given as mean ± SEM as the results of ordinary one-way ANOVA followed by Tukey’s multiple comparison test

The presence of the BBB, a highly selective semipermeable border between the capillaries and the brain is a major obstacle that dramatically hinders the drug delivery. Available reports are controversial for integrity of BBB in CPZ fed mice. Here, to ensure that DNC is able to pass intact BBB we used an *in-vivo* imaging system. Our observation indicated that DNC is detectable in brain region of mice with intact BBB within 1-2 h after being injected intraperitoneally (12.5 mg/kg). We also could detect DNC fluorescence emission in harvested brain, under UV-light excitation beam. However, with the same exposure time and gain settings, no significant fluorescent signal was indicated in mouse that did not receive DNC injection (Figure1, B 1-2). 

*The Effect of DNC on CPZ-induced changes in the OLLC composition of CC*

CPZ demyelination is induced by oligodendrocytes toxic death, rather than an attack on the myelin directly. Reactivation of astrocytes, microglia accumulation, mitochondrial injury, and release of ROS have been suggested as a major driving force of OLs apoptosis. Thus, approaches that modulate these mechanisms potentially provide a new range of therapeutic strategies. Traditionally, OLs death and demyelination are viewed as targets of an uncontrolled autoimmune inflammatory response. But, a number of clinical and experimental experiments have reported that OLs injury/death seems to occur prior to disease process and initiate the formation of demyelinating lesions (1, 31, 32). However, it is suggested that protection of OLs against injury results in protection against MS progression and symptoms (33, 34). On the basis of curcumin-mediated cytoprotection against oxidative stress and inflammation (35, 36), here we investigated that protective effect of DNC on OLLCs in context of CPZ toxicity. Thus, the numbers of OLLC was investigated by IHF against Olig2, as specific marker of these cells. In CPZ + PBS group, CC contained reduced numbers of olig2^+^ cells compared to the control; however, the number of Olig2^+^ cells was significantly higher in CPZ+DNC treated mice compared to PBS treated group (Figures1 C-D). As CPZ is known to induce specific death of OLLC, this result suggested that DNC exhibited protective effect on these cells against CPZ. Another explanation might be the inductive effect of DNC on migration of CNS resident oligodendrocyte progenitor cells (OPCs) toward demyelinated lesions. 

Available studies have reported the neuroprotective effect of curcumin or its nano-formulations in different CNS disorders. This effect is well-established to be at least in part via anti-inflammatory and anti-oxidative stress property of curcumin. I one available study, curcumin is reported to protect pre-oligodendrocytes from infection-derived apoptosis. This protection is suggested to be associated with suppression of nitric oxide synthase (iNOS) and NADPH oxidase (NOX) (37). However, to best of our knowledge no available study has reported protective effect of curcumin or its nano-formulations in demyelinating lesions. 

*The Effect of DNC on astrocytes and microglia composition of CC*

Astrocytes reactivation and microglia accumulation are well-established features of demyelinated lesions in MS. Both of these events lead to production of large amount of ROS, leading to oxidative injury followed by mitochondrial dysfunction. Thus, they play significant role in OLs injury and their dysfunction.

**Figure 2:**
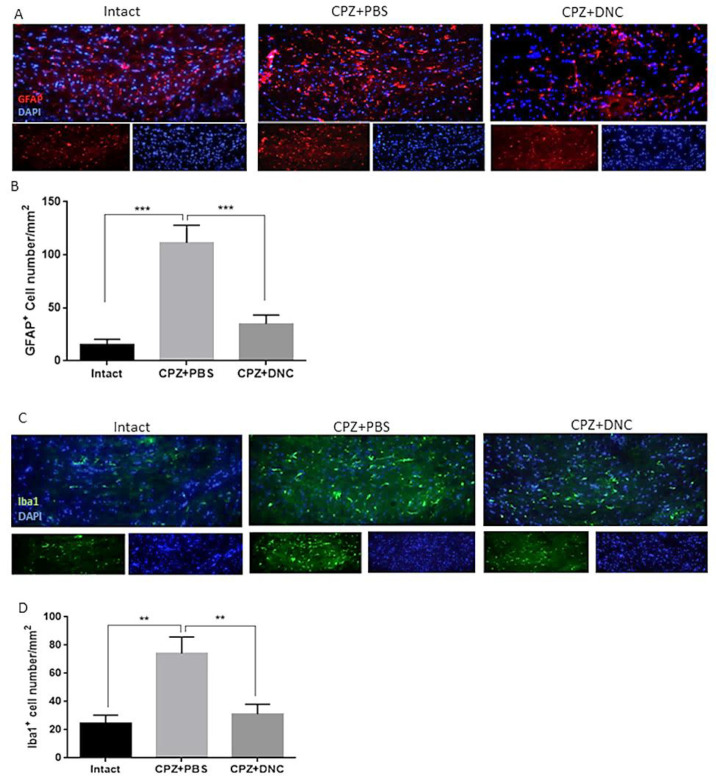
DNC treatment suppressed accumulation and activation of astrocytes and microglia in CC of CPZ treated mice. (A) Representative image of IHF of brain sections for GFAP, marker of reactive astrocytes. (B) Comparing the number of GFAP^+^ cells in intact group with CPZ + PBS group showed that CPZ treatment lead to significant increase in number of reactive astrocytes in CC; however, DNC treatment along with CPZ feeding, lead to significantly lower number of GFAP^+^ cells in CC. (C) Representative image of IHF of brain sections for Iba1, marker of microglia. (D) Comparing the number of Iba1^+^ cells between intact mice and CPZ + PBS group showed that CPZ intake lead to significant accumulation of microglia in CC; however, significant decrease in Iba1+ cells in DNC treated group showed suppressive effect of this compound on microglia activation by CPZ. N = 27 brain sections from 3 mice, per experiment group. Values are given as mean ± SEM as the results of ordinary one-way ANOVA followed by Tukey’s multiple comparison test

As CPZ-induced demyelination is accompanied by activation and accumulation of astrocytes and microglia, mimicking a central feature of MS, we investigated the effect of DNC on number of astrocytes and microglialcells within CC by IHF against GFAP^+^ and Iba1^+^ as markers of these cells, respectively. As shown in Figures 2 A-B, the CPZ+PBS group showed significantly increased number of GFAP^+^ cells compared to the control group; however, DNC treatment was associated with significantly lower number of GFAP^+^ cell in CC, when being compared with PBS treated mice. Astrogliosis formation is one of the consequences of myelin damage; thus, this finding suggested inhibitory effect of DNC on glial scar formation. Accumulation of reactive astrocytes at the site of CNS injury is a seminal feature of damaged or diseased CNS. As, this impairs OLLS survival and remyelination capacity; we suggest that DNC may exhibit its protective effect on OLLCs by reduction of astrocytes’ activation.

As mentioned, CPZ model coincide with Iba-1 + microglia migration toward CC. Accumulated microglia release inflammatory and cytotoxic mediators, including ROS and iNOS. This contributes to the formation of inflammatory microenvironment, cell death and neurological dysfunction. OLs are the main target cells in such cytotoxic condition. It is reported that the number of Iba-1+ microglia is inversely proportional to OLs, suggesting that the accumulation of inflammatory microglia is closely related to myelin loss. As we have previously showed anti-oxidative stress and anti-inflammatory properties of DNC, here we investigated the direct effect of this compound on accumulation microglia, as main producers of inflammatory mediators. Our results showed that even though the number of microglial cells was significantly higher in CPZ+PBS treated groups, CPZ feeding accompanied with DNC treatment lead to significantly lower number of these cells in CC (Figure 2C-D).

Pathological process including inflammatory response and glial scar formation are major causes of progression of CNS- injuries and inhibition of repair. Curcumin has been showed to curcumin promotes spinal cord repair through inhibiting glial scar formation and inflammation (38). Under the influence of many relevant factors, microglia cells and astrocytes are activated in features of MS lesions. This will lead formation of dense glial scar and production of excessive amount of inflammatory mediators and extracellular matrix components, such as chondroitin sulfate proteoglycan. This process leads to death of myelination cells and inhibition of remyelination. Our previous study, along with the other reports has indicated neurotrophic property of curcumin and its nano-formulation, in CNS damage context (9). As a neurotrophic environment contributes to the survival and regeneration of OLLCs in CPZ-induced demyelination, we suggest that DNC promoted OLLCs survival/proliferation by providing a support to inhibit their apoptosis by CPZ. 

*The effect of DNC on myelination index of CC following acute CPZ intake*

**Figure 3:**
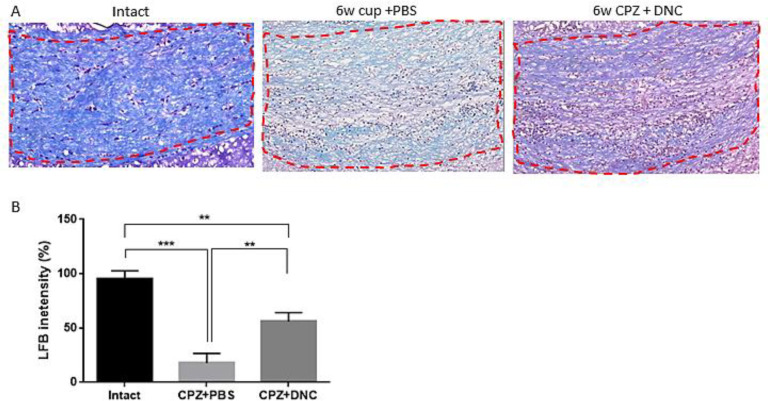
DNC treatment showed protective effect on myelin content of CC during CPZ intake. (A) Representative images for LFB staining results.( B) Evaluation of myelination index by analyzing LFB intensity indicated that even though in intact mice the myelin sheets showed normal, multi-layered and compact structure 6 weeks treatment with CPZ and PBS lead to disorganized myelin composition with significantly lower intensity, compared to intact mice. But, in group that received DNC along with CPZ intake the degree of myelination was significantly higher than PBS treated group. The degree of myelin, however, was still lower in DNC group, compared to intact mice. n = 27 brain sections from 3 mice, per experiment group. Values are given as mean ± SEM as the results of ordinary one-way ANOVA followed by Tukey’s multiple comparison test

Considering protective effect of DNC on OLLC, along with its repressive effect on astrocyte and microglia activation, hypothetically we assumed that DNC protects against toxic myelin damage by CPZ. In this regard, we evaluated the protective effect of DNC on myelin content of CC. It is well-established that after 5-6 weeks CPZ intake, the CC region losses its myelin content and an acute demyelination occurs. Hereby using LFB staining, we observed a normal myelin structure in the intact group (Figure 3A). However, the animals treated with CPZ and PBS (CPZ+PBS) exhibited a significant decrease in LFB staining, characterized by light and a disorganized myelin sheet in CC. Comparing the semi-quantitative results of LFB intensity showed that in the animals treated with CPZ and DNC (CPZ + DNC group), the degree of LFB staining was significantly higher than that in the CPZ + PBS group (Figure 3A-B). 

**Figure 4:**
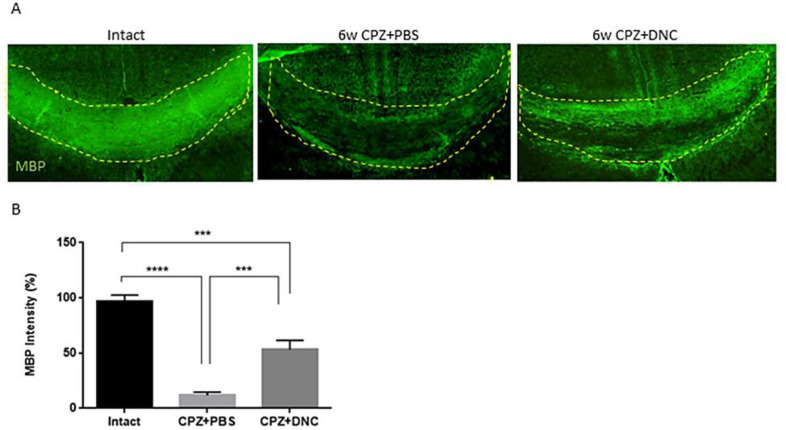
DNC treatment showed protective effect on MBP content of CC after CPZ intake. A) Representative images from brain sections IHF for MBP, marker of main components of myelin sheath. B) Analysing the intensity of MBP in CC of mice from intact and CPZ+PBS group showed that 6 weeks CPZ intake lead to significant depletion in myelin content of CC; however, the degree of myelination was significantly higher in DNC treated group compared to PBS treated one. n = 27 brain sections from 3 mice, per experiment group. Values are given as mean ± SEM as the results of ordinary one-way ANOVA followed by Tukey’s multiple comparison test

In order to further confirm the protective effect of DNC on myelin content of CC against CPZ, along with LFB, immunostaining for MBP as a marker of myelination was performed. Our findings showed that DNC treatment lead to higher degree of myelin intensity CC, compared to CPZ+PBS group (Figure 4).

Several lines of evidences have indicated that OLs death and disruption of myelin integrity are associated with many pathological and neurodegenerative symptoms of MS. Thus, protecting myelin or myelinating cells at early stages of MS is likely to be of clinical relevance. Current therapies for MS are designed to modulate or suppress the inflammatory processes of the disease, but provide limited benefit for survival of myelinating cells and preservation of myelin sheaths. Even though, a number of studies has shown the anti-inflammatory, antioxidant, immunomodulatory, anti-astrogliosis properties of curcumin in MS (39, 40), here we indicated a new feature of curcumin, as a nano-formulation, that protects myelin forming cells from apoptotic death by CPZ induced oxidative stress, apparently due to inhibition of inflammatory, non-permissive microenvironment induced by microglia and astrocytes. Although the potential underlying molecular mechanisms of these effects by DNC remains to be elucidated, the results from our study suggest DNC as a promising compound to protect or promote myelin index in context of demyelinating conditions. However, future studies are required to confirm the real benefits of DNC for the management of demyelinating diseases. 

**Table 1:** List of antibodies used in current study

**Target**	**source**	**Labled cells**	**Company**
Olig2	Rabbit	Oligodendroglial lineage cells	Abcam, ab9610
GFAP	Rabbit	Reactive astrocytes	DAKO, Z0334
Iba1	Rabbit	Microglia	sc-32725
MBP	Mouse	Mylin	Abcam, ab40390
